# Extract of Malt and Phosphorus Compounds in the Treatment of Phthisis

**Published:** 1878-05

**Authors:** Elwood C. Lester

**Affiliations:** Philadelphia, Pa.


ART. III.—*Extract of Malt and Phosphorous Compounds in the Treatment of Phthisis.* By Elwood C. Lester, M. D., Philadelphia, Pa.

Perhaps, with the exception of pneumonia, there is no disease the treatment of which is in a more chaotic state than phthisis. There is not a single remedy, out of the hosts of specifics which have been offered, that really is accepted by the profession. Cod liver oil, which of all remedies has the most advocates, in a large per cent. of cases disorders the digestive functions, and thereby inflicts a larger degree of injury than benefit. The bark of the wild cherry with sulphuric acid will unquestionably do a greater aggregate of good. Naptha is almost a thing of the past, and the phosphorous compounds are involved in the confusion of statements made by friends and foes, so that it is difficult to arrive at a satisfactory estimate of their value. As is well known, in 1855 Churchill took the ground that the deficiency of phosphorous in the formula of hypo-phosphorous acid in its oleo-nitrogenous association with alkaline and mineral bases, was the determining cause of phthisis. We all know that his theory as well as his remedy—the hypo-phosphites of lime and soda—were enthusiastically received, but he promised too much, and his extravagances in statement, I think, have in a large measure injured the reputation of his agents. A large per cent. of the failures which have followed the employment of the hypo-phosphites have resulted from relying upon them exclusively, to the omission of the adjuvants, and with an utter disregard of existing pathological conditions. The theory advanced by Dr. Polk, that by the deranged and deficient nerve influence transmitted from the medulla oblongata and base of the brain nutrition is disordered, and butyric acid is generated, is probably correct. Roussin's experiments have given the uniform results—butyric acid masks all the tests for phosphorous. With the strumous dyspepsia (as Sir James Clark termed the digestive disorder in which the acid condition is created), it is obvious that the acid condition of the chyle will destroy all the physiological and therapeutical properties of the hypo-phosphites, consequently to give hypo-phosphites without neutralizing this acid condition is the height of folly, for it must be evident that they will be held too firmly in the grasp of the butyric acid, will be changed into phosphates, and escape by the kidneys and bowels without partaking in the vital processes of the system. If this statement is founded in good philosophy, it must appear that nothing but disappointment must follow the usual manner of giving the hypo-phosphites. It seems that the butyric acid has less control of the calcium hypo-phosphite, and I have no doubt that nearly all the good that accrued from the employment of the compound syrup was given by this salt. The writer regrets not being familiar with the ideas of Churchill, further than that in 1855 he conceived the idea that consumption was in consequence of deficiency of the hypophosphites in the system, ; that during the two succeeding years he tried the hypo-phosphites as remedial agents, and in July, 1857, reported his results in thirty cases to the Academy of Medicine, Paris. I am not sure whether he realized the antagonism between his remedy and the acid condition of the stomach and duodendum; if he did, and counteracted it, the writer can in a large degree accept his views. The writer had but just begun the practice of his profession in his native State (Massachusetts), when Churchill's remedy was announced. He tried it in a large number of cases; in a few the result was truly gratifying, but in a large per cent. no advantage was derived. So, with him, it fell out of favor, and by 1876 had ceased to be employed. During that year I began giving a patient five grains of the hypo-phosphite of lime and a tablespoonful of extract of malt three times a day. The patient, a lady of thirty, had been going from bad to worse under previous treatment. Cod liver oil, given in various forms and numerous vehicles had sickened her stomach; iron and quinia had seemed to predispose to hemorrhage, and to aggravate the cough; but ere two weeks had elapsed from the time she began taking the malt and hypo-phosphite of lime an improvement began, and continued until the lady seemed restored. I have since tried the same practice in about twenty cases, and so uniformly satisfactory has been the result that I have no inclination to try any other mode of treatment.

In infantile phthisis I have had satisfactory results accrue from the use of "Wheat Phosphates," made according to Tilbury Fox's formula, and "Organic Phosphates" made by Ashmead's formula. The therapeutics of phthisis may thus be summed up; A rich, nutritious diet, out door exercise, bowels regular, and rely on the extract of malt and hypo-phosphite of lime to improve digestion, emulsify the fatty portion of the food so as to obviate butyric fermentation and restore the missing phosphorous elements. Gallic acid, ergot and sulphuric acid to relieve hæmorrhage, also night sweats; sulpho-carbolate of sodium, salicine and quinia for chills, and hectic fever; wild cherry bark, hydrocyanic acid, cimicifuga, sanguinaria and morphia for the cough; tincture of iron, dilute phosphoric acid, quinia and strychnia, or the syrup of the Phosphates of Iron, Quinia and Strychnia as a tonic in anæmia, and the wine of beef and iron in the last stages of the disease to preserve the strength and support life, and render the last weeks of existence more comfortable.

This wine of beef contains all the nutrient principles of the flesh and blood of the cow, and in my experience, which has yet been limited to a few cases, it throws every other pharmaceutical preparation of beef in the shade. I am quite enthusiastic as to its value.

